# Design and Evaluation of a Multiplexed Assay to Assess Human Immunogenicity Against Humira®

**DOI:** 10.1208/s12248-020-00487-4

**Published:** 2020-08-03

**Authors:** Matthew Alleyn, Kristin Closson, Adam Gentile, Nathan Gulbis, Christopher Taylor, Paul Rhyne

**Affiliations:** 1 Immunologix Laboratories, 4710 Eisenhower Blvd, Building D, Tampa, Florida 33634 USA; 2 Bill & Melinda Gates Medical Research Institute, One Kendall Square Building 600 Suite 6-301, Cambridge, Massachusetts 02139 USA

**Keywords:** bead array, Humira®, immunoassay, immunogenicity, Luminex®, multiplex

## Abstract

The use of biologic-based therapeutics has revolutionized our ability to treat complex diseases such as cancer- and autoimmune-related disorders. Biologic-based therapeutics are known to generate anti-drug immune responses or immunogenicity in clinical patients which can lead to altered pharmacokinetics, decreased drug efficacy, and unwanted adverse clinical events. Assays designed to detect and assess anti-drug immune responses are used to help monitor patients and improve drug safety. Utilizing a tiered approach, screening assays are developed first to identify patients that are potentially positive for anti-drug-specific antibodies. Patients that screen positive are subjected to additional tiers of testing that include a confirmation assay to confirm the presence of expected anti-drug-specific antibodies, a titer assay to assess relative levels of anti-drug-specific antibodies, and, depending on the drug’s mechanism of action or concerns of adverse clinical reactions, further characterization such as drug neutralization and anti-drug antibody isotyping. This tiered approach can prove to be detrimental to clinical samples from exposure to multiple cycles of testing, freeze thaws, and repeated handling by lab personnel. Multiplexing some of these assays together may streamline the characterization of anti-drug immune responses and help reduce the repeated usage of clinical samples. In this study, we combined a screening assay and anti-drug isotyping assays into one multiplexed assay using the Luminex® xMAP® Technology. The multiplexed assay was developed and validated to meet the FDA recommended guidelines for immunogenicity assessments. These results show that multiplexed assays perform comparably to industry standards. This study should encourage labs to explore the use of multiplexing immunogenicity assays to characterize anti-drug antibody responses quickly, with less repeat testing and reduced sample handling.

## INTRODUCTION

The pharmaceutical industry has generated many novel therapeutics that are typically protein-based molecules derived from biologic sources. However, the use of therapeutic protein products often initiates immune responses against the therapeutic protein that can potentially lead to adverse clinical events in patients (1–3). An early example is the use of recombinant human erythropoietin to treat anemia in patients with chronic renal failure. Most of the patients that were treated with recombinant human erythropoietin responded well resulting in increased red blood cell production. Unfortunately, some of these patients developed pure red blood cell aplasia that was resistant to further treatment with recombinant erythropoietin. Subsequent analysis revealed these patients were producing antibodies against the recombinant erythropoietin that neutralized its activity, which also bound to the endogenous form of the glycoprotein. This resulted in a severe downregulation of erythropoiesis and the development of pure red blood cell aplasia (2, 3). Another example is cetuximab, which is a chimeric mouse/human IgG_1_ monoclonal antibody that binds to epithelial growth factor. Cancer patients treated with cetuximab sometimes had hypersensitivity reactions at the injection site attributed to the presence of pre-existing IgE antibodies against specific glycosylation sites present on the drug. These IgE antibodies were likely generated from previous exposure to certain grasses, pollen, and animal tissues expressing similar glycosylation modifications (4). Hypersensitivity reactions have been observed in multiple sclerosis patients treated with a humanized drug (natalizumab) which targets integrin alpha 4. Some of these patients developed rashes and had shortness of breath due to IgE-mediated hypersensitivity from the drug (5). Hemophilia A patients have been reported to develop IgG antibodies against recombinant factor VIII which diminished the effectiveness of the therapy (6). Other examples of unwanted clinical adverse events attributed to antibodies include treatment with erythromycin (7), infliximab (8), enzyme replacement therapies (9,10), and rapid drug clearance in patients that affect the pharmacokinetic profile of the drug (11). The industry and regulatory agencies have responded to these instances with strategies to detect and characterize anti-drug antibodies to improve drug safety (1).

Strategies for evaluating immunogenicity have been developed to detect the presence of anti-therapeutic antibodies or anti-drug antibodies (ADA) in drug development. These strategies rely upon a tiered approach involving a panel of immunogenicity assays. The first tiered assay is a qualitative screening assay used to determine the presence of ADA in a given sample. If a positive result is generated, the sample is subsequently tested in a confirmation assay that uses competitive binding with the drug to confirm the presence and drug-specificity of ADA (12,13). Confirmed positive samples may be subject to further immunogenicity assays including antibody titer assessment, drug neutralizing assays (14), cross-reactivity assays, and isotyping assessments (1). The sum of the data generated from these assays is used to assess and characterize ADA responses, and to help make informed decisions on drug safety.

There are a few disadvantages to using tiered approaches, such as subjecting the samples to multiple freeze-thaw cycles, repeated analysis in different assays, and limited sample volume especially with pediatric samples. Multiplexing some of these assays together may help alleviate these consequences by maximizing utilization of the sample to generate faster, more detailed results. There are many different combinations of immunogenicity assays that could be multiplexed together, such as screening, cross-reactivity, specificity, and isotyping. Published examples of immunogenicity assays being multiplexed together exist and are readily available. McCutcheon et al*.* developed a multiplexed ADA isotyping assay in cynomolgus monkeys treated with Raptiva (humanized monoclonal antibody to CD11a) (15). The study showed the ability to measure IgG, IgM, IgA, and IgE ADA antibodies in a single assay format. Granath et al. developed an ADA isotyping assay using beads from a commercial Luminex® isotyping kit (16). In this study, they immunized mice with a biologic drug and used isotype-specific bead sets to capture antibodies. Isotypes of drug-specific antibodies were assessed using biotin-labeled drug and streptavidin-phycoerythrin (SAPE). The authors showed that ADA titers from different isotypes could be measured using a multiplexed approach. While these studies have effectively shown that multiplexed assays are versatile and useful tools, the study in this paper describes the development of a multiplexed multi-tiered assay that combined screening and isotyping ADA assays. The isotyping portion of the assay was expanded to include the main classes and subclasses of immunoglobulins including IgG_1_, IgG_2_, IgG_3_, and IgG_4_, IgM, IgE, IgA_1_, and IgA_2_ (17,18). There are many technologies that can be used for multiplexing including Mesoscale Discovery (19), LC/MS (20), Biacore (21), Quanterix, Gyros, Imperacer, Squidlife Technologies, and Genalyte Maverick System (22). We selected the Luminex FLEXMAP 3D® system which offers flexibility to add/remove analytes in the lab (up to 500 bead sets), ease of coupling antibodies to beads, availability of reagents and instruments from many commercial vendors (MilliporeSigma Corporation, St. Louis, MO; Bio-Rad Laboratories, Hercules, CA; R&D Systems Inc., Minneapolis, MN), and use to support clinical studies (23–25). This study describes the development, validation, and application of a multiplexed multi-tiered immunogenicity assay that combines a screening assay and isotyping analysis together. We selected the human monoclonal therapeutic Humira® as our experimental drug given its well described immunogenicity profile (26–30). Humira® (adalimumab) is a recombinant human IgG_1_ antibody that binds to TNFα and is approved for treatment of rheumatoid arthritis, psoriatic arthritis, Crohn’s disease, and other inflammatory diseases. In summary, the data presented here demonstrate that it is feasible to combine different immunogenicity tiers together in a multiplexed assay format. The assay performed well overall and with further optimization, could be used to conduct immunogenicity assessment of ADA in a clinical trial setting.

## MATERIALS AND METHODS

### Anti-Human Antibodies and Supporting Reagents

Mouse anti-human IgG_1_ (#9054-01), mouse anti-human IgG_2_ (#31-7-4), mouse anti-human IgG_4_ (#9200-01), and mouse anti-human IgE (#9250-01) were obtained from Southern Biotechnology (Birmingham, AL). Mouse anti-human IgG_3_ (#05-3600) was obtained from Invitrogen (Carlsbad, CA) and mouse anti-human IgM (#555856) and mouse anti-human IgA (#555886) were obtained from BD Biosciences (San Jose, CA). Secondary detection reagents including biotin-mouse anti-human kappa light chain (#555790) and biotin-mouse anti-human lambda light chain (#555794) were obtained from BD Biosciences (San Jose, CA). R-Phycoerythrin-conjugated streptavidin (#016-110-084) and R-Phycoerythrin-conjugated donkey F(ab’)_2_ anti-mouse IgG (H + L) (#715–116-150) were obtained from Jackson ImmunoResearch (West Grove, PA).

### Human Isotype Control Antibodies

Human IgG1 Kappa (#0151K-01), human IgG3 lambda (#153L-01), human IgM lambda (#185L-01), and human IgA kappa (#155K-01) isotype control antibodies were obtained from Southern Biotechnology (Birmingham, AL). Human IgG2 kappa (HCA193), human IgG4 kappa (HCA195), human IgE kappa (HCA190), and the ADA positive control antibody human IgG1 anti-Adalimumab (Humira®) (HCA204) were obtained from Bio-Rad Laboratories (Hercules, CA).

### Antibody Conjugation to Luminex Beads

Capture antibodies were covalently conjugated to MagPlex® carboxylated bead sets (Luminex Corporation, Austin, TX) using manufacturer’s instructions. Briefly, stock solutions of each bead set were vortexed and sonicated for 20 s prior to coupling. From the stock vial, 2.5 × 10^6^ beads from each bead set were removed from, placed into a microcentrifuge tube, and washed by placing the bead mixture in a microcentrifuge tube magnetic separator for 1 min. The supernatant was aspirated, and the remaining beads were resuspended in deionized water via vortex and sonication for 20 s. The beads were washed via the magnetic separator and resuspended in 80 μL of 0.1 M sodium phosphate, pH 6.2 via vortex and sonication. Ten microliters of a 50 mg/mL freshly prepared Sulfo-NHS solution and 50 μL of a 10 mg/mL freshly prepared (1-ethyl-3-(3-dimethylaminopropyl)carbodiimide hydrochloride) (EDC) solution (Thermo Fisher Scientific, Waltham, MA) were added to the bead suspension followed by gentle vortexing. The beads were incubated at ambient room temperature in the dark for 20 ± 2 min, with a brief vortex every 10 min. The beads were washed twice via magnetic separator with 250 μL of 50 mM 2-(N-morpholino) ethanesulfonic acid (MES, Thermo Fisher Scientific, Waltham, MA) pH 5.0, then resuspended in 100 μL of 50 mM MES via vortex and sonication for 20 s each. 12.5 μg of the antibodies to be coupled were added to a final volume of 400 μL 50 mM MES (pH 5.0). This entire volume was added to the respective tubes containing the distinct bead regions, gently vortexed, and allowed to incubate in the dark at ambient room temperature for 2 h via rotation. The beads were then separated via magnet and the supernatant aspirated. The beads were resuspended in 500 μL of 1% BSA in PBS-T (PBS-TB), 1% BSA, 0.1% polysorbate-20, and 0.05% sodium azide (PBS-TB) via vortex and sonication for 20 s each. The beads were then allowed to incubate in the dark at ambient room temperature for 1 h via rotation. After incubation, the bead counts were approximated using a hemocytometer and then diluted down to a working concentration of 1000 beads/μL. Coupled beads were stored at 4 °C in the dark for up to 3 months.

### Biotinylation of Humira®

**Fig. 1:**
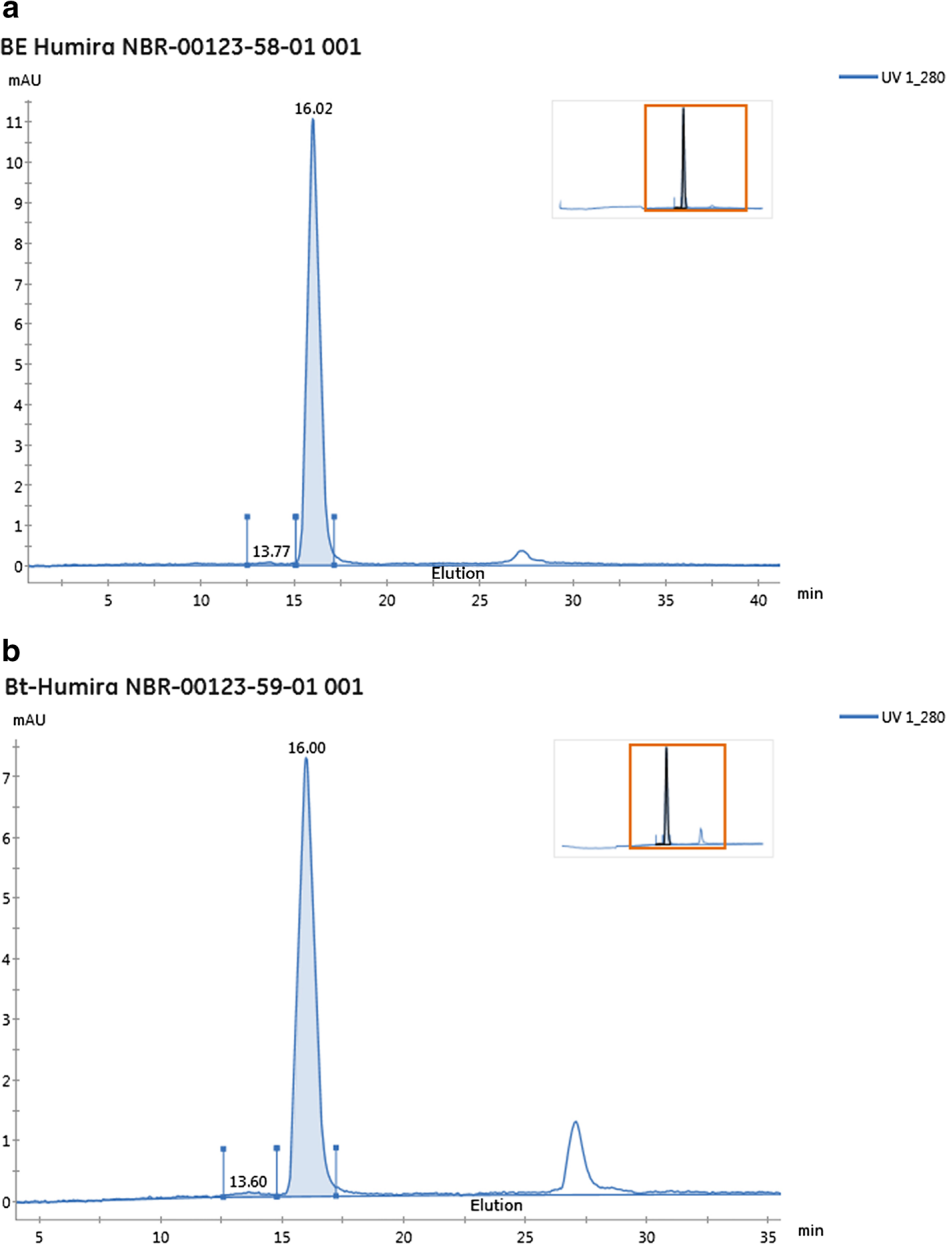
Biotinylation and characterization of Humira®. The figure shows the analysis of Humira® on a Superdex 200 Increase 10/300GL column equilibrated in PBS (0.75 mL/min flow rate) before (**a**) and after labeling with biotin (**b**). The high molecular weight species was 1.2% in the unlabeled preparation and 1.92% in the biotin-labeled preparation

Twenty-five microliters of Humira® lot 42497XDD4 (AbbVie Inc.) at 47.8 mg/mL was assessed for degradation and aggregation prior to biotin labeling using FPLC-based analysis and size exclusion chromatography (SEC) prior to labeling with biotin. Figure 1 shows a chromatogram from the unlabeled Humira® analysis and shows a predominately monomeric form of Humira® with a minimal presence of aggregates (less than 2%) and a lack of multiple smaller protein species suggesting no degradation. Humira® was diluted to 10 mg/mL using phosphate-buffered saline (× 1 PBS) and buffer exchanged to remove any unwanted components that could interfere with the biotin coupling process by using a 7 K MWCO Zeba column (Thermo Fisher Scientific, Waltham, MA) following the manufacturer’s recommendations. The protein concentration after buffer exchange was determined using Pierce BCA Protein Assay Kit (Thermo Fisher Scientific, Waltham, MA) following the manufacturer’s recommendations. Buffer exchanged Humira® was diluted to 2.0 mg/mL PBS and conjugated using a × 10 molar excess of EZ-Link Sulfo-NHS-LC Biotin (Thermo Fisher Scientific, Waltham, MA). During conjugation, the material was incubated on a tube rotator for approximately 2 h protected from light at room temperature. Removal of unbound biotin was performed using a 40 K MWCO Zeba column (Thermo Fisher Scientific, Waltham, MA) equilibrated with 25 mM histidine buffer, pH 6.0, and the final preparation of biotinylated Humira® was stored in 25 mM histidine buffer, pH 6.0 at − 70 °C until further use. Samples of buffer exchanged Humira® and the final biotinylated Humira® product were evaluated for aggregation using a Superdex 200 Increase 10/300 GL column on an Äkta Pure 25 FPLC instrument (GE Healthcare Life Sciences, Pittsburgh, PA). The chromatograms from before and after biotin labeling are shown in Fig. 1 and suggest no aggregation before or after labeling. The final concentration of the biotin-Humira® preparation was determined using BCA Protein Assay Kit (Thermo Fisher Scientific, Waltham, MA) following the manufacturer’s recommendations. Biotin incorporation was confirmed using Pierce Biotin Quantitation Kit (Thermo Fisher Scientific, Waltham, MA) following manufacturer’s recommendations and yielded a biotin incorporation rate of 3.8 biotins per molecule. The final biotinylated Humira® was diluted to 1.0 mg/mL in 25 mM histidine, pH 6, divided into 50 μL aliquots, and stored at nominal − 70 °C.

### Surface Testing of Conjugated Beads

The efficacy of the coupling procedure for the Ig antibody bead sets was determined using R-Phycoerythrin labeled secondary antibodies purchased from Jackson ImmunoResearch Laboratories (West Grove, PA). Briefly, 50 μL of each bead set to be tested (1000 beads/μL) were mixed in PBS-TB to a final volume yielding 50 beads/μL per bead set. Fifty microliters of this bead mixture was added to each well of a 96-well plate. Surface testing for bead sets conjugated with mouse anti-human Fc antibodies was done by adding 50 μL of PE-anti-Mouse IgG (H + L) diluted in PBS-T to the manufacturer’s working concentration to each well. The 96-well plate was incubated in the dark at ambient room temperature with shaking at approximately 450 RPM for 1 h. The plate was then washed using a Biotek 405 magnetic plate washer (Biotek Instruments, Winooski, VT) set to perform a cycle consisting of three washes of 150 μL of PBS-TB per well. Each well was resuspended in 150 μL PBS-TB before immediately analyzing the plate on a Luminex FLEXMAP 3D instrument obtained through R&D Systems Incorporated (Minneapolis, MN). Surface testing of Humira® conjugated beads was done by adding 50 μL of biotin-labeled anti-human kappa light chain to the manufacturer’s working concentration and incubated in the dark at ambient room temperature with shaking at approximately 450 RPM for 1 h followed by a wash step. One hundred microliters of 1:50 SAPE diluted in PBS-T was added to each well and the 96-well plate was incubated in the dark at ambient room temperature with shaking at approximately 450 RPM for 30 min. The plate was then washed using a Biotek 405 magnetic plate washer (Biotek Instruments, Winooski, VT) set to perform a cycle consisting of three washes with 150 μL of PBS-TB per well. Each well was resuspended in 150 μL PBS-TB before immediately analyzing the plate on a Luminex FLEXMAP 3D instrument obtained through R&D Systems Incorporated (Minneapolis, MN).

### Assay for Binding of Isotype Controls

The ability of anti-Human Ig bead sets to bind their respective human IgG_1_, IgG_2_, IgG_3_, IgG_4_, IgA, IgM, and IgE isotype control antibodies was assessed as follows. All the bead sets were mixed in PBS-TB to a final concentration of 50 beads/μL/bead set. Fifty microliters of this solution was added to each well of a 96-well plate. Fifty microliters of PBS-TB containing 1000 ng/mL of each isotype control antibody was added to each well. The beads were incubated in the dark at ambient room temperature while shaking at approximately 450 RPM for 1 h, followed by several wash cycles using a magnetic plate washer set to perform a cycle consisting of three washes of 150 μL of PBS-TB per well. The beads were resuspended in 50 μL PBS-TB and mixed with 50 μL of biotin-labeled anti-human kappa light chain at 0.125 μg/mL or biotin-labeled anti-human lambda light chain at 1.0 μg/mL (BD Biosciences, Woburn, MA) and incubated in the dark at ambient room temperature with shaking at approximately 450 RPM for 1 h. The plate was washed with the magnetic plate washer and each well resuspended in 50 μL PBS-TB. Fifty microliters of SAPE (Jackson ImmunoResearch, West Grove, PA) at 1:100 dilution was added to each well. The plate was incubated in the dark at ambient room temperature with shaking at approximately 450 RPM for 30 min. The plate was washed, and each well was resuspended in 150 μL in PBS-TB before immediately analyzing the plate on a Luminex FLEXMAP 3D.

### Enrichment of Humira® Reactive ADA from Serum Samples

Serum samples were enriched for Humira® reactive ADA using biotin-labeled Humira® prior to being analyzed in the Luminex assays. Briefly, 50 μL of serum samples pre-diluted 1:8 in PBS-TB were diluted 1:2 with 600 mM acetic acid and allowed to incubate at ambient room temperature with shaking at approximately 450 RPM for 10–20 min. One hundred microliters of 2 μg/mL biotinylated Humira® in 370 mM Tris was added to the acidified samples and allowed to incubate at ambient room temperature with shaking at approximately 450 RPM for 30–60 min. Pierce High Binding Streptavidin-Coated Plates (Thermo Fisher Scientific, Waltham, MA) were washed with PBS-T and residual wash buffer tapped out before transferring 90 μL of neutralized and biotinylated samples to the streptavidin plate. The samples were then incubated in the streptavidin plate at ambient room temperature with shaking at approximately 450 RPM for 50–79 min. After sample incubation, the streptavidin plate was again washed with PBS-T and residual wash buffer removed. Eighty microliters of 100 mM acetic acid was then added to each well and incubated at ambient room temperature with shaking at approximately 450 RPM for 10–25 min. The samples were immediately used in the Luminex assay.

### Analysis of Samples with Luminex Assay

A master mix of capture beads was prepared in PBS-TB to give 50 beads/μL per bead set. Fifty microliters of this mix was added to each well in a 96-well plate. The plate was washed using a magnetic plate washer and the wells were resuspended in 60 μL of either 1% bovine IgG or 150 mM Tris for screening or isotyping assays, or 10 μg/mL Humira® in 1% bovine IgG or 150 mM Tris for confirmatory assays. Sixty microliters of the acidified samples from the enrichment of Humira® reactive ADA step described above were added to the plate and incubated overnight at 5°C with shaking at approximately 450 RPM. After this incubation, the plate was washed with a magnetic plate washer set to perform a cycle consisting of three washes of 150 μL of PBS-TB per well. The beads were resuspended in 50 μL PBS-TB and mixed with 50 μL/well of biotin-Humira® followed by a 1-h incubation at ambient room temperature in the dark while shaking at approximately 450 RPM. The plate was washed with the magnetic plate washer’s 150 μL cycle, followed by dispensing 50 μL PBS-TB to resuspend the beads. Finally, 50 μL of SAPE diluted 1:100 in PBS-TB was added to each well and incubated at ambient room temperature in the dark with shaking at approximately 450 RPM for 30 min. The plate was washed and each well resuspended in 150 μL PBS-TB before immediately analyzing the plate on the FLEXMAP 3D instrument.

### Screening and Confirmatory Cut Point Determinations

Fifty individual serum samples from normal healthy subjects and 20 serum samples from Humira® naive rheumatoid arthritis (diseased) subjects (BioIVT, Westbury, NY) were analyzed in the multiplexed screening and isotype assay 6 times across multiple days with multiple analysts. The screening ratios (mean sample signal / mean negative control signal) were obtained from all cut point runs, log transformed, and pooled for analysis. The confirmatory assay inhibition ratios were calculated using Humira® (10 μg/mL) spiked samples vs. unspiked samples (mean drug spiked sample signal/mean unspiked sample signal). Inhibition ratios were calculated from all cut point runs, log transformed, and pooled for analysis. JMP statistical software (SAS Institute Incorporated, Cary, NC, software version 14) was used to compare healthy and diseased screening population distributions for each of the assays in the multiplexed panel. The mean and standard deviations of the two populations were determined and evaluated for statistical variation using the one-way ANOVA. The populations were considered statistically similar if the *F* value obtained from the ANOVA demonstrated 95% confidence (*p* ≥ 0.05). If the screening populations were determined statistically similar, the data sets from each population were pooled for both the screening and confirmatory cut point determination. Otherwise, separate healthy and diseased cut points were calculated for each screening and confirmatory assay.

The Explore Outliers function of JMP was then used to identify any outliers (values greater than the upper quartile + 3 × inter-quartile range and values less than the lower quartile – 3 × inter-quartile range). All outliers identified were removed from all subsequent calculations. After the removal of outliers, the median response, median absolute deviation, and skewness of the population were assessed. The absolute value of the skewness was used to assess the distribution of the populations. If the absolute value of the skewness was less than or equal to 1, the population was considered normally distributed and the cut point was calculated using the robust parametric approach outlined below. If the population was not normally distributed, the cut point was calculated non-parametrically as the 95th (screening) or 99th (confirmatory) percentile of the log transformed ratios.

$${\displaystyle \begin{array}{c}\mathrm{Screening}\ \mathrm{cut}\ \mathrm{point}=10\hat{\mkern6mu} \left(\mathrm{Median}\ \mathrm{Response}+\left(1.645\times \left(1.4826\times \mathrm{median}\ \mathrm{absolute}\ \mathrm{deviation}\right)\right)\right)\\ {}\mathrm{Confirmatory}\ \mathrm{cut}\ \mathrm{point}=100\times \left(1\hbox{--} \left(10\hat{\mkern6mu} \left(\mathrm{median}\ \mathrm{response}\hbox{--} \left(2.33\times \left(1.4826\times \mathrm{median}\ \mathrm{absolute}\ \mathrm{deviation}\right)\right)\right)\right)\right)\end{array}}$$

## RESULTS

### Selection of Drug Target and Assay Design

**Fig. 2:**
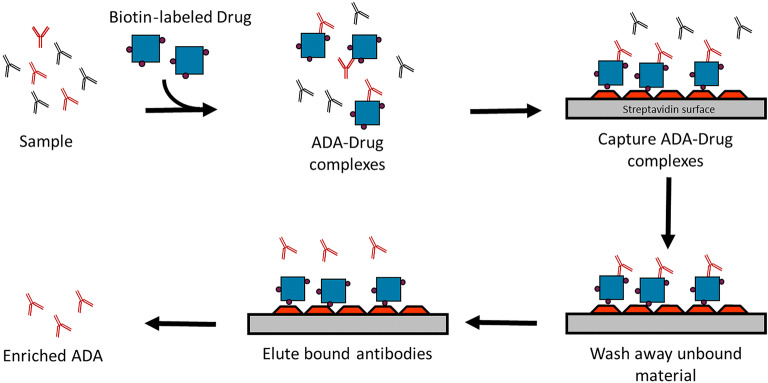
Enrichment of anti-Humira® reactive antibodies from samples. The figure shows use of biotin-labeled Humira® to capture Humira® reactive ADA from the sample in solution. The ADA biotin-Humira® complexes were then captured on streptavidin-coated plates, washed, and the ADA released into solution using 100 mM acetic acid. The ADA enriched sample was neutralized with 150 mM Tris buffer and analyzed in the multiplexed assay

**Fig. 3:**
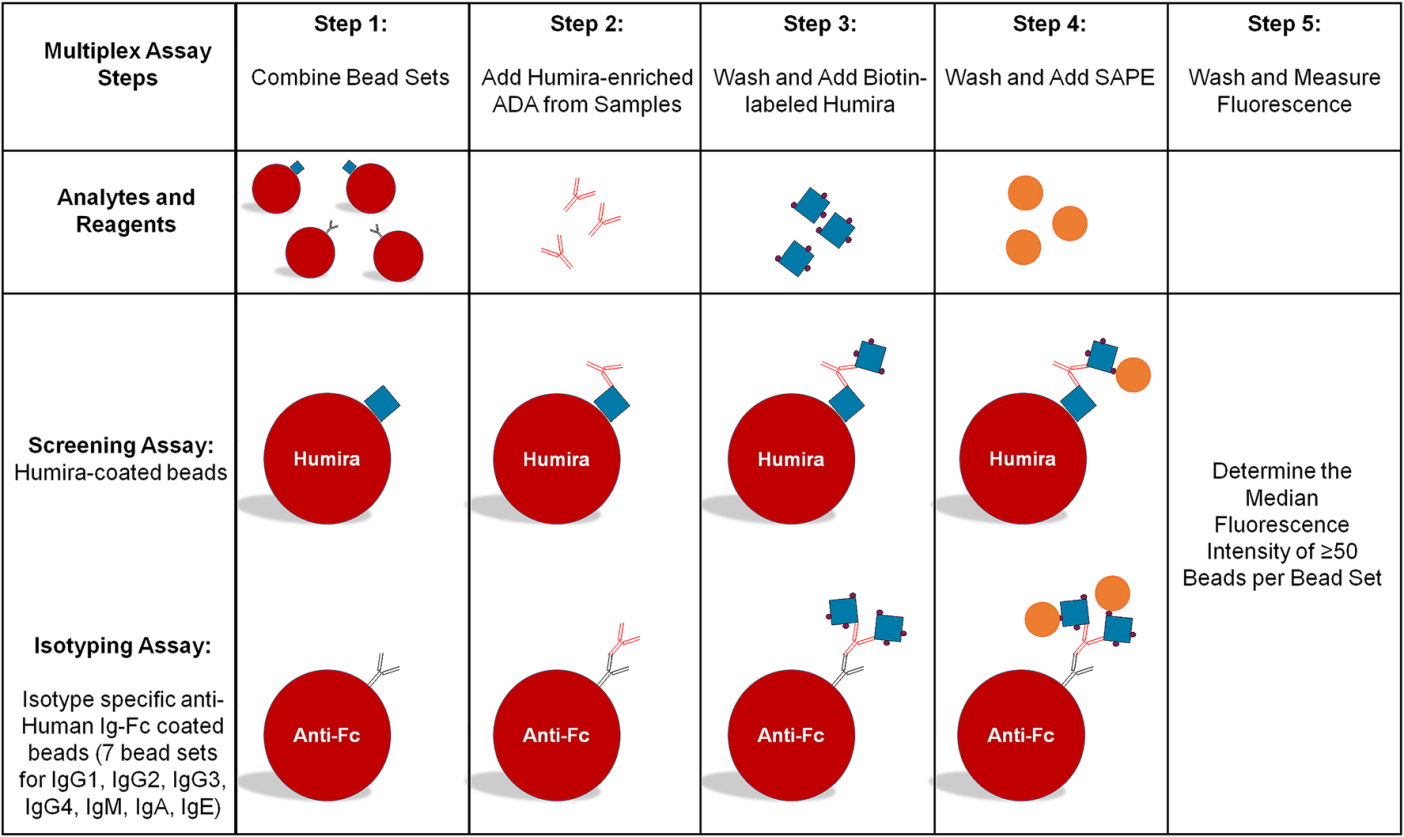
Multiplexed screening and isotyping assay. The figure depicts the steps of the combined multiplexed assay. Enriched Humira® reactive ADA (Fig. 2) were added to the combined bead sets and incubated to allow the ADA to bind accordingly. The beads were washed and mixed with biotin-labeled Humira® followed by the addition of SAPE. The captured ADA on each bead set were measured via PE fluorescence on a Luminex FLEXMAP 3D instrument

The purpose of this study was to combine multiple immunogenicity-tiered assays together in a multiplex format. The Luminex xMAP platform was selected for this study due to its open architecture, ease of covalent coupling of capture antibodies to beads, and established industry presence. The technology allows for discrete assays to be conducted on the surface of polystyrene microspheres or beads, which are distinguished from one another via a unique ratio of fluorescent dyes incorporated into the beads. Immunoassays are performed on the bead surface and detected by a fluorochrome such as phycoerythrin (PE). Multiplexing works by mixing the bead sets together and analyzing the mixture in a Luminex instrument that distinguishes the bead sets from one another using multiple lasers or LEDs. The instrument uses gating to distinguish single beads from beads that are clumped together during the analysis and calculates the median PE fluorescence intensity of each bead set from at least 50 individual beads per set. Humira® was selected as a model system since there are many commercially available reagents, human serum samples from normal healthy individuals, and serum from rheumatoid arthritis patients that have been treated with Humira®. Since all serum samples contain high levels of non-Humira® specific antibodies which would interfere with the isotyping assessment, we used biotin-labeled Humira® to enrich Humira® reactive ADA from samples prior to analysis (Fig. 2). The isotyping assay format was done using isotype-specific antibodies coupled to Luminex beads that captured their respective isotype-specific antibodies from the sample followed by detection with biotin-labeled Humira® and SAPE (Fig. 3). The screening assay was performed using a modified bridging format where Humira® was conjugated directly to the bead surface and used to capture Humira® specific ADA from the sample. Detection of captured ADA was done via biotin-labeled Humira® which would bind to the open paratopes on the captured ADA to complete the Humira:ADA:biotin-Humira complex. SAPE was added to enable detection of the complex by the instrument (Fig. 3).

### Covalent Conjugation of Antibodies to Luminex Bead Sets

The measurement of human isotypes required a panel of isotype-specific capture antibodies, each coupled to a unique Luminex bead set. Monoclonal isotype-specific antibodies were purchased from commercial sources (see materials and methods section for details) and covalently coupled to Luminex MagPlex magnetic carboxylated beads using an EDC conjugation protocol as described in the materials and methods section. After the conjugation, we confirmed the presence of the antibodies on the bead surface by conducting a “surface test.” This was done by mixing the various conjugated bead sets together with a phycoerythrin anti-mouse IgG (heavy and light chain reactive) antibody to the bead mixture. The data in Table I show the average measured median fluorescence intensity (MFI) from at least 50 beads/set per well. It is important to point out that the expected MFI levels from the surface testing are arbitrary and is common for monoclonal antibodies to give varied levels of MFI when comparing them to one another. Generally, an MFI between 10,000 and 25,000 indicates a high level of antibody on the bead surface and suggests a successful conjugation. The IgE-conjugated bead set yielded an MFI of 6410, which was lower than the other antibodies, but high enough to confirm the presence of the antibody on the bead surface. Surface testing of Humira®-coated beads was done with biotin-labeled anti-human kappa light chain antibody followed by incubation with SAPE. The surface test showed a high level of Humira® on the bead surface (26,923 MFI) suggesting a successful conjugation.

**Table I:** Confirmation of Antibody Coupling to the Surface of Luminex MagPlex Beads

	IgG_1_	IgG_2_	IgG_3_	IgG_4_	IgM	IgA	IgE	Humira®
Anti-mouse IgG	28,808	22,634	23,467	17,822	17,023	14,265	6410	58
Anti-human kappa light chain	458	170	14	67	12	45	13	26,923

The bead sets were mixed with PE-anti-mouse IgG (H + L) to confirm the conjugation of mouse capture antibodies to bead surface. Confirmation of Humira® conjugated to beads was done using biotin-anti-human kappa light chain followed by SAPE. The data show the average MFI from three replicate wells

### Assessment of Assay Specificity and Detection of Multiple Human Antibody Isotypes

All the bead sets had measurable levels of their respective capture antibody and were ready to be tested for the ability to capture their specific human isotype antibody. We assessed the specificity of each bead set by mixing each bead with a panel of human isotype control antibodies. The ability of each bead set to capture its specific human antibody isotype control antibody was assessed via detection with biotin-labeled anti-human light chain antibodies (either anti-kappa or -lambda light chain separately) followed by SAPE. Table II-A shows the binding of human isotype control antibodies that contained a kappa light chain and Table II-B shows the binding of human isotype control antibodies that contained a lambda light chain.

**Table II:** Specificity Assessment of Multiplexed Bead Sets

Bead set	IgG_1_	IgG_2_	IgG_3_	IgG_4_	IgM	IgA	IgE	Humira®
A. Binding of kappa light chain isotype controls								
Human IgG_1_ kappa	3431	20	23	31	17	124	21	28,276
Human IgG_2_ kappa	69	6232	27	38	22	55	17	27,686
Human IgG_3_ lambda	61	21	52	32	19	286	19	28,623
Human IgG_4_ kappa	63	2136	22	22,080	18	60	22	28,021
Human IgM lambda	56	23	18	33	27	70	21	27,443
Human IgA kappa	51	23	25	32	28	23,698	19	27,004
Human IgE kappa	54	26	21	41	19	69	10,946	27,525
B. Binding of lambda light chain Isotype controls								
Human IgG_1_ kappa	31	27	24	36	21	93	21	57
Human IgG_2_ kappa	20	32	20	38	20	56	22	55
Human IgG_3_ lambda	22	35	17,771	40	21	206	20	61
Human IgG_4_ kappa	21	31	23	43	17	57	20	52
Human IgM lambda	19	32	22	43	13,085	349	25	50
Human IgA kappa	27	31	25	49	21	56	26	52
Human IgE kappa	22	28	23	36	19	64	20	58

The bead sets were incubated with various human isotype control antibodies followed by biotin-anti-human kappa light chain (A) or biotin-anti-human lambda light chain (B) and SAPE. The table shows the average MFI from three separate wells

The data in Table II show that each of the human isotype control antibodies were successfully captured to their respective capture bead set and generated a high MFI value. We noted that the IgG_4_ isotype control gave a high MFI on the anti-IgG_4_ capture bead (MFI of 22,080) and a low MFI on the IgG_2_ capture bead set (MFI of 2136) suggesting some non-specific binding. Each of the commercial antibody certificates of analysis had data that tested the specificity of their product to its specific isotype, though some of the monoclonal antibodies including the IgG_4_ isotype control were generated by recombinant technology and possibly contained epitopes that were not as isotype-specific as claimed. Overall, the data in Table II show that each of the isotype-specific antibodies were highly specific and gave good MFI levels to perform effectively in a multiplex assay.

### Specificity for Humira® ADA

There are many commercial kits on the market that perform isotype testing. These typically capture isotype-specific antibodies from the sample using anti-human capture antibodies. The assays typically utilize a labeled anti-human light chain antibody as a detection reagent. Although these isotype-specific kits work well, they do not measure antibodies that are specific to Humira® (or any other biologic). Thus, the use of commercial isotyping kits would not work for immunogenicity assessment for any specific biologic. Therefore, we designed the multiplexed assay to detect ADA that are reactive to Humira®. This was done by mixing all the bead sets together (including the bead set conjugated with Humira® itself) with the sample, followed by biotin-labeled Humira® and SAPE. This ensures that, the ADA detected in our multiplexed assay were reactive to Humira®.

The specificity assessment of the multiplexed assay was done using the commercially available positive control human IgG_1_ kappa anti-Humira® antibody. We were unable to source human anti-Humira® specific positive controls for the other isotypes. The human IgG_1_ kappa anti-Humira® positive control antibody was added to the multiplexed assay as shown in Table III-A. The results show the background MFI on the IgG_1_ capture bead set was 1053 and less than 100 MFI for all the other bead sets. The higher background MFI for the IgG_1_ bead set was likely due to the binding of biotin-Humira® in conjunction with SAPE. However, the positive control antibody generated an MFI of 18,777 on the IgG_1_ capture bead set and an MFI of 2841 on the Humira® bead set. The positive control antibody yielded MFI levels comparable to background levels on all the other bead sets.

**Table III:** Specificity of Bead Sets

	IgG_1_	IgG_2_	IgG_3_	IgG_4_	IgM	IgA	IgE	Humira®
A. Positive control assessment								
Background in assay buffer	1053	21	20	19	15	20	10	60
Human IgG_1_ anti-Humira®	18,777	102	19	31	13	13	14	2841
B. Assignment of cut point factors for screening and confirmatory assays								
Screening assay	IgG_1_	IgG_2_	IgG_3_	IgG_4_	IgM	IgA	IgE	Humira®
Normal cut point factor	1.44	1.29	1.39	1.36	6.49	2.36	1.15	1.31
Rheumatoid arthritis cut point factor	1.44	1.35	1.39	1.36	6.49	1.95	1.13	1.73
Confirmatory assay	IgG_1_	IgG_2_	IgG_3_	IgG_4_	IgM	IgA	IgE	Humira®
Normal cut point factor	84.9	38.2	42.6	39.1	37.5	35.6	18.2	50.4
Rheumatoid arthritis cut point factor	84.9	27.6	42.6	39.1	37.5	38.1	18.2	76.1

(A) Shows the binding of the positive control antibody to the different bead sets. Assay buffer preparations with or without the positive control (2500 ng/ml) were mixed with the bead sets followed by biotin-Humira® and SAPE. (B) Shows the calculated screening assay cut points as determined from using 50 normal healthy individual samples or 10 rheumatoid arthritis serum samples. The confirmatory cut points were done using the same samples spiked with Humira® at 10 μg/mL. The data was determined from six independent repeats of each sample over multiple days and analysts

### Assignment of Screening Cut Point and Isotyping Cut Points

Screening assays that follow regulatory guidelines utilize a statistically derived cut point to determine if a sample is potentially ADA reactive or not. This is typically done via analysis of a panel of human samples from drug naïve individuals which would represent ADA negative values. There also needs to be consideration for disease states since there are instances where the background levels in the assay will be different for samples taken from individuals with disease versus other diseases or normal healthy individuals. Thus, our approach was to use a set of samples from mainly normal, healthy drug naive individuals in addition to a set of samples from diseases that are often treated with Humira® (e.g., psoriasis or rheumatoid arthritis).

We used a panel of normal healthy individuals obtained from commercial sources. Individuals who had not been treated with any biologic molecule, especially Humira®, were requested. Samples were also obtained from individuals with rheumatoid arthritis that had not been treated with Humira®. Following the recommendations from the FDA’s guidance for assessment of immunogenicity, these samples were analyzed 6 times over the course of multiple days with multiple analysts. The data set generated from these runs were analyzed for analytical and biological outliers, then used to generate screening cut points with a 5% false positive rate. A cut point was generated for the screening assay and for each of the bead sets in the isotyping panel. The results are shown in Table III-B. The screening assay was converted to a confirmatory assay by adding in excess unlabeled Humira® at 10 μg/mL to the assay. A confirmatory cut point was subsequently calculated with a 1% false positive rate in comparison to an unspiked sample. The confirmatory cut point calculations are shown in Table III-B.

### Sensitivity and Selectivity Assessment of Screening and Isotyping Assays

Using the FDA guidelines for immunogenicity assessment, we assessed both selectivity and sensitivity of the screening assay and for the IgG_1_ portion of the isotyping assay. We could not assess specificity nor sensitivity of the other portions of the isotyping assay due to lack of specific positive controls for each of the other isotypes. The sensitivity of the screening, confirmatory, and IgG_1_ isotyping assays were done by spiking in various levels of the human IgG_1_ anti-Humira® positive control into normal, healthy, and Humira® naive individual samples. The concentration of spiked ADA that crossed the assigned cut point represented the sensitivity. The results are shown in Table IV and suggest the sensitivity of the screening assay (using Humira® beads) was 287 ng/mL and the confirmatory assay yielded a sensitivity of 403 ng/mL. The IgG_1_ isotyping assay gave a sensitivity of 902 ng/mL. We also performed some initial drug tolerance experiments where we tested the drug tolerance at 50 μg/ml of Humira® with the PC concentrations ranging from 1000 to 5000 ng/ml. The results showed drug tolerance of at least 50 μg/ml with the screening assay and the IgG1 isotyping assay (data not shown).

**Table IV:** Sensitivity Assessment of Screening and Confirmatory Assays

IgG_1_	Run 1	Run 2	Run 3	Run 4	Run 5	Run 6	Mean ng/mL
Sensitivity (ng/mL)	254	481	895	2326	881	577	902
Humira®	Run 1	Run 2	Run 3	Run 4	Run 5	Run 6	Mean ng/mL
Screening sensitivity (ng/mL)	NA	91.6	199	482	190	472	287
Confirmatory sensitivity (ng/mL)	122	120	225	315	480	1156	403

Sensitivity of the screening assay and the confirmatory assay was calculated from 6 runs using the Human IgG_1_ anti-Humira® positive control over a wide range (500 ng/mL to 3.91 ng/mL) on IgG_1_ and Humira® beads. The confirmatory assay used samples spiked with 10 μg/mL of Humira®

Selectivity was performed by spiking in the human IgG_1_ anti-Humira® positive control at 5000 ng/mL (estimated to be equivalent to a high-level PC) into normal healthy and Humira® naive individual samples. The results from the selectivity experiment are shown in Table V. The screening assay and the IgG_1_ isotyping assay gave positive results from all spiked samples (normal healthy and rheumatoid arthritis samples) which were positive in the confirmation assay (italicized values in Table V). There were a small number of individuals that generated positive results for other isotypes which were confirmed positive. Individual 4 from the normal healthy group was positive for IgG_3_ ADA, individual 3 from the rheumatoid arthritis disease group was positive for IgE, and individual 5 from the rheumatoid arthritis disease group was positive for IgG_3_.

**Table V:** Analysis of Positive Control Spiked Normal Healthy Individual and Rheumatoid Arthritis Samples

Normal healthy serum	IgG_1_	IgG_2_	IgG_3_	IgG_4_	IgM	IgA	IgE	Humira®
Screening Cut Point	1.44	1.29	1.39	1.36	6.49	2.36	1.15	1.31
1	*1.70*	1.27	1.23	0.81	2.54	1.15	1.10	*8.04*
2	*1.63*	1.07	1.32	0.90	1.23	1.15	1.00	*6.91*
3	*1.90*	1.13	1.64	1.10	1.46	1.92	1.00	*9.00*
4	*1.85*	0.93	*1.68*	0.90	1.31	1.15	0.90	*8.85*
5	*1.71*	1.07	1.68	1.00	1.31	1.69	1.10	*7.91*
Rheumatoid arthritis serum	IgG_1_	IgG_2_	IgG_3_	IgG_4_	IgM	IgA	IgE	Humira®
RA screening Cut Point	1.44	1.35	1.39	1.36	6.49	1.95	1.13	1.73
1	*2.14*	1.03	0.82	1.25	4.11	4.18	1.04	*10.91*
2	*1.31*	1.33	1.12	1.20	54.95	5.55	0.96	*9.10*
3	*2.41*	1.03	0.88	1.15	1.63	1.45	*1.22*	*13.32*
4	*2.52*	1.21	1.18	1.15	1.37	1.45	0.87	*15.76*
5	*2.37*	1.09	*1.70*	1.20	1.53	1.82	1.13	*13.67*

Data show results from five individual normal healthy serum and five individual rheumatoid arthritis serum samples spiked with 5000 ng/mL of human anti-Humira® positive control. The results in italics show screen positive samples that confirmed positive in the confirmatory assay. RA, rheumatoid arthritis; *CP*, cut point

### Analysis of Rheumatoid Arthritis Samples from Individuals Treated with Humira®

The multiplexed assay was used to analyze individual serum samples from rheumatoid arthritis subjects who have been treated with Humira®. The data in Table VI show that 5 of the 10 samples had ADA positive screening results in the screening assay. However, only 2 of those samples confirmed positive in the confirmatory assay. The isotyping analysis correlated with the screening assay with the same two samples having confirmed presence of one or more ADA isotypes. Sample 4 confirmed positive in the screening assay and confirmed positive for IgG_3_ ADA. Sample 6 confirmed positive in the screening assay and confirmed positive for IgG_1_, IgG_2_, and IgG_3_. There were samples that gave high signals in the screening assay that did not confirm positive (samples 1, 3, and 7).

**Table VI:** Analysis of Serum from Rheumatoid Arthritis Subjects Being Treated with Humira®

Rheumatoid subjects	IgG_1_	IgG_2_	IgG_3_	IgG_4_	IgM	IgA	IgE	Humira®
Screening Cut Point	1.44	1.29	1.39	1.36	6.49	2.36	1.15	1.31
1	2.21	1.38	29.70	1.24	7.35	2.60	1.21	7.31
2	0.92	0.97	0.98	0.99	0.93	1.00	1.04	0.94
3	1.52	1.04	1.89	1.06	3.59	1.01	1.05	3.62
4	2.97	1.09	*19.98*	1.13	6.25	1.69	1.01	*7.05*
5	0.82	1.04	1.36	1.11	1.16	1.15	1.12	0.92
6	*4.07*	*2.08*	*7.03*	1.36	0.90	1.46	1.14	*10.40*
7	1.22	1.11	1.37	1.11	4.88	1.58	1.10	5.15
8	1.26	0.93	0.97	0.96	0.91	0.98	1.03	0.95
9	0.69	0.88	0.91	0.88	0.77	0.80	0.92	0.74
10	0.88	0.90	1.07	1.05	1.26	1.10	1.06	1.01

Data show results from 10 individual samples from rheumatoid arthritis subjects that have been treated with Humira®. Data that are italicized represent screen positive samples that were confirmed positive. *CP*, cut point

## DISCUSSION

The results presented in this study demonstrate that immunogenicity assays can be developed and multiplexed together using the Luminex xMAP Technology. We were able to show that a screening assay can be performed simultaneously with ADA isotyping assays in a multiplexed format. Although the assays were not fully optimized, they did perform well with regard to the FDA recommendations for immunogenicity assays (1). The overall sensitivity of the screening assay did not reach 100 ng/mL, per FDA recommendations; however, there are several strategies to improve the sensitivity that could be used to further optimize the assay. The number of beads/set per well in this study was 2500 beads/well; altering the levels of beads has been shown to impact the assay sensitivity. Increasing the number of beads per well can increase the binding capacity of the assay, leading to increased upper levels of sensitivity. Likewise, decreasing the number of beads per well may increase the lower limits of sensitivity. The concentration and coupling conditions used to couple the capture reagents to the beads influences their ability to capture analytes. Other isotype-specific antibodies may prove to be more efficient capture reagents which would improve sensitivity as well. The ADA enrichment step we used using biotinylated Humira® could also be further optimized to increase the amount of ADA enriched from the sample. This includes optimizing the amount of biotinylated Humira® used in the well, incubation time and temperature, and the use of streptavidin-coated beads in lieu of streptavidin-coated plates. Finally, the Luminex FLEXMAP 3D instrument utilizes the fluorescence of phycoerythrin to generate the assay signal. There are further calibration conditions that have been used that may increase the fluorescence, which can also impact the assay sensitivity.

The positive control antibody used in this study was a recombinant human IgG_1_ monoclonal antibody purchased from a commercial source. This positive control worked well in this study and showed positive signals in the IgG_1_ isotyping assay. Most immunogenicity assays rely on positive controls derived from hyperimmunized animals when drug-specific human monoclonal antibodies are not available. In these circumstances, additional bead sets coated with species-specific antibodies could be placed into the isotyping portion of the assay to allow for positive controls to be incorporated. There are strategies to generate additional controls by conjugating the positive control antibodies derived from animals with the Fc portion of different human isotypes. The resulting conjugate would retain the drug-binding portion of the positive control and the conjugated human Fc molecule would bind to the isotype capture bead set. Thus, these modified positive controls would be expected to generate signals in the isotyping assays described in this study.

Table III shows data from experiments evaluating the specificity of the different bead sets. Humira® itself is a human IgG_1_ kappa antibody which did bind to the IgG_1_ capture bead set and generate an MFI of 1053 (Table III-A). However, the positive control antibody generated a much higher MFI (18777) on the IgG_1_ capture bead. One explanation for this increased MFI is the total amount of biotin-Humira® in the final immune complex. In the case of biotin-Humira® by itself, only two biotin-Humira® antibodies would be bound for each anti-IgG_1_ capture antibody on the bead surface. In comparison, the number of biotin-Humira® antibodies after the positive control antibody is much higher. There would be two positive control antibodies bound to each anti-IgG_1_ capture antibody on the bead surface which subsequently binds four biotin-Humira® antibodies (two for each positive control antibody), generating a much higher MFI. It is also possible that the capture monoclonal anti-IgG_1_ antibody we used in this study may not bind well to biotin-Humira®. Other human monoclonal therapeutics used in this assay format would be expected to have background signals with the biotinylated drug alone on the corresponding anti-Fc bead set. In anticipation of this, one strategy to help mitigate this would be to insert a blocking step with a non-specific isotype control antibody between the sample incubation step and the addition of biotinylated drug. This would fill the remaining binding sites on the beads with non-specific antibodies and help mitigate the binding of biotin-drug to the beads. There is also a need to optimize the amount of biotinylated drug used in the assay to maximize the detection of drug-specific ADAs while minimizing the background MFI due to direct binding to the corresponding anti-Fc bead set. This will also help improve the overall sensitivity of the isotyping assay for the corresponding anti-Fc bead set.

We did observe high screening cut points in the isotyping assays for IgM (cut point of 6.49) and IgA (cut point of 2.36) in comparison to the other isotypes. IgM typically exists in a pentamer form and IgA exists mainly in the monomeric form in serum but there are low levels of dimeric, trimeric, and tetravalent IgA present in serum (most of the dimeric forms of IgA are found in mucosal secretions) (17,31–33). Furthermore, rheumatoid arthritis patients often have auto-antibodies including IgM and IgA that bind to the Fc portion of IgG (34,35). Therefore, the combination of the multivalent nature of IgM and IgA combined with their possible rheumatoid factor activity may have contributed to the high IgM and IgA cut points in the screening assays in comparison to the other isotyping assays.

Traditionally, immunogenicity screening assays are based on a bridging format where drug conjugates labeled with a detection molecule (such as biotin) are mixed in solution with the clinical sample. Immune complexes formed between the drug conjugates and the ADA present in the sample are subsequently captured on a streptavidin surface. This approach maximizes the availability of drug epitopes to be accessible to ADA; however, bridging formats rely on the ability of ADA to have both paratopes available to generate a signal (10). There has been concern over the ability of bridging assays to detect IgG_4_ ADA that have switched chains with other IgG_4_, which makes them monomeric for a given antigen (36). In these instances, monomeric IgG_4_ would not generate a positive signal in bridging assays. The isotyping assay format utilized in this study does not require both paratopes to be available in the enrichment step nor the isotyping assays, and is therefore uniquely able to detect monomeric IgG_4_ ADA present in serum.

This study demonstrates the ability to multiplex different immunogenicity assays together. The Luminex FLEXMAP 3D platform offers 500 different bead sets. Thus, it is plausible that other immunogenicity assays could be multiplexed in addition to the ones described here. One example would be cross-reactivity assessment where additional bead sets could be conjugated with related or non-related molecules. Epitope mapping is also possible by conjugating peptides with overlapping sequences on different bead sets. Additionally, reactivity to carbohydrate modifications, reactivity to polyethylene glycol (PEG), degradation products derived from the drug, or related drugs such as biosimilars could also be incorporated into the multiplex assay. In summary, multiplexing of immunogenicity assays is feasible and should be further explored in the industry. This approach has the potential to generate an abundance of additional information on ADA responses while reducing the amount of labor to generate additional data without sacrificing sample volume or risking sample integrity via excess handling.

## CONCLUSIONS

In this study, we developed and validated a multiplexed assay that combined two immunogenicity assays on the Luminex FLEXMAP 3D system. The multiplex assay demonstrated performance comparable to industry standards, and with further optimization could be effectively used to conduct a multiplexed immunogenicity assessment in a clinical trial setting. Laboratories should explore the use of multiplexed immunogenicity assays for rapidly characterizing anti-therapeutic antibody responses. Multiplexed analyses such as these described for the Luminex system can decrease the need for repeat testing of clinical samples and help ensure sample integrity with reduced handling and fewer freeze-thaw cycles.
